# Cathepsin S attenuates endosomal EGFR signalling: A mechanical rationale for the combination of cathepsin S and EGFR tyrosine kinase inhibitors

**DOI:** 10.1038/srep29256

**Published:** 2016-07-08

**Authors:** Chien-Chang Huang, Cheng-Che Lee, Hsiao-Han Lin, Jang-Yang Chang

**Affiliations:** 1 National Institute of Cancer Research, National Health Research Institutes, Tainan, Taiwan, ROC; 2 Division of Hematology and Oncology, Department of Internal Medicine, National Cheng Kung University Hospital, College of Medicine, National Cheng Kung University, Tainan, Taiwan, ROC; 3 Institute of Basic Medical Sciences, College of Medicine, National Cheng Kung University, Tainan, Taiwan, ROC

## Abstract

EGF-mediated EGFR endocytosis plays a crucial role in the attenuation of EGFR activation by sorting from early endosomes to late endosomes and transporting them into lysosomes for the final proteolytic degradation. We previously observed that cathepsin S (CTSS) inhibition induces tumour cell autophagy through the EGFR-mediated signalling pathway. In this study, we further clarified the relationship between CTSS activities and EGFR signalling regulation. Our results revealed that CTSS can regulate EGFR signalling by facilitating EGF-mediated EGFR degradation. CTSS inhibition delayed the EGFR degradation process and caused EGFR accumulation in the late endosomes at the perinuclear region, which provides spatial compartments for prolonged EGFR and sustained downstream signal transducer and activator of transcription 3 and AKT signalling. Notably, cellular apoptosis was markedly enhanced by combining treatment with the EGFR inhibitor Iressa and CTSS inhibitor 6r. The data not only reveal a biological role of CTSS in EGFR signalling regulation but also evidence a rationale for its clinical evaluation in the combination of CTSS and EGFR tyrosine kinase inhibitors.

Epidermal growth factor receptors (EGFRs), which are transmembrane receptors with tyrosine kinase activity, play a crucial role in the switch control between tumour cell survival and death. EGFR expression was reported to increase in various tumours including bladder, colon, ovarian, and kidney cancers; non-small cell lung carcinoma and glioma; ovarian and pancreatic cancer as well as breast tumors and head and neck squamous cell carcinoma^1^. Through the binding of different ligands, EGFR signalling cascades regulate various biological processes, including cell proliferation, division, differentiation, angiogenesis, and metabolism. Upon ligand binding, EGFR dimerisation undergoes autophosphorylation on multiple tyrosine (Y) residues within the cytoplasmic domain of EGFR, such as EGFR-Y992, -Y1045, -Y1068, and -Y1173. The tyrosine phosphorylation of EGFR subsequently leads to the recruitment of diverse adaptor proteins for activating downstream signal transduction molecules, including AKT, ERK1/2, signal transducer and activator of transcription 3 (STAT3), and p38 mitogen-activated protein kinases (MAPK). Moreover, appropriate temporal and spatial localisations of activated EGFR complexes tightly regulate the different signalling cascades^2^,^3^,^4^. In a previous study, prolonged EGFR signalling from late endosomes in the peripheral region caused both sustained ERK and p38 signalling, whereas continuous EGFR signalling from late endosomes in the perinuclear region only caused sustained ERK signalling^5^. Although EGFR signalling is required for cell survival and proliferation^6^, prolonged EGFR signalling was reported to promote cell apoptosis^2^. EGFR signalling primarily begins from the plasma membrane, continuously transmits signalling from early and late endosomes, and is finally attenuated in lysosomes through proteolytic degradation^4^,^7^. Thus, endocytosis of activated receptors is a crucial mechanism for negatively regulating receptor signalling. Notably, Tjelle *et al*. observed that late endosomes can degrade nearly 80% of endocytic substrates but lysosomes that have the highest concentration of lysosomal enzymes do not exert higher degradative abilities^8^. This also implied that certain enzymes are proteolytically active in late endosomes (approximately pH 5–6) and might play a crucial role in the regulation of EGFR signalling attenuation.

Cathepsins, which are cysteine proteases of the papain family, have been typically implicated in the major proteolytic function of lysosomes^9^,^10^. Most cathepsins show an optimal proteolytic activity at an acidic pH; however, cathepsin S (CTSS) is highly active and stable in both acidic and neutral microenvironments. The CTSS activity profiles revealed a bell-shaped pH activity, at a pH optimum of 6.5^10^, which is similar to the pH profile of an endosomal microenvironment. However, few data are available on the intracellular role of CTSS in the regulation of receptor signalling attenuation. CTSS is synthesised as a preproenzyme of 331 residues, comprising a signal peptide of 16 residues and a proregion of 98 residues; cysteine residue 25 located in the catalytic pocket of CTSS is responsible for the substrate specificity and catalysis. Thus, the replacement of cysteine residue 25 with alanine can impair the proteolytic ability of CTSS^11^,^12^,^13^. In addition to its biological role in inflammation, increased CTSS expression has been reported in various malignant tumours^14^,^15^,^16^,^17^,^18^,^19^,^20^; CTSS activity has been proposed to play a crucial role in tumour progression^21^,^22^,^23^. CTSS can be secreted in the extracellular region and be proteolytically active for the remodelling of an extracellular matrix, such as collagen, elastin, fibronectin, and laminin^24^,^25^,^26^, and has therefore been suggested to affect cell migration, invasion, proliferation, and tumour angiogenesis. Considering its crucial role in tumour progression, CTSS has been suggested as a potential therapeutic target for multiple types of cancer^27^,^28^. Pharmacological inhibition or molecular silencing of CTSS not only reduce the spread of malignant cells, HUVEC tube formation, and tumour vascularisation^23^,^28^,^29^ but also rapidly induce tumour autophagy, oxidative DNA damage, and consequent cell apoptosis^30^,^31^,^32^,^33^,^34^.

We previously reported that the inhibition of CTSS activities or reduction of CTSS expression induces EGFR activation and downstream ERK/MAPK signalling cascades. Moreover, both CTSS and EGFR hold potential as therapeutic targets for cancer treatment. Thus, the relationship between CTSS and EGFR appears to be crucial and warrants further study. In this study, we demonstrated that CTSS is responsible for the regulation of EGFR signalling. CTSS inhibition delays the EGFR degradation process and causes the perinuclear distribution of accumulated EGFR within late endosomes. Furthermore, continuous EGFR signalling from late endosomes contributes to sustained downstream AKT and STAT3 but not ERK1/2 or p38 signalling cascades. Moreover, an endosomal accumulation of the activated EGFR does not increase tumour cell apoptosis but further increases tumour cell survival. However, a combination of the CTSS inhibitor 6r and the EGFR tyrosine kinase inhibitor Iressa synergistically promotes tumour cell apoptosis.

## Results

### CTSS is involved in the EGFR degradation process

**Figure 1:**
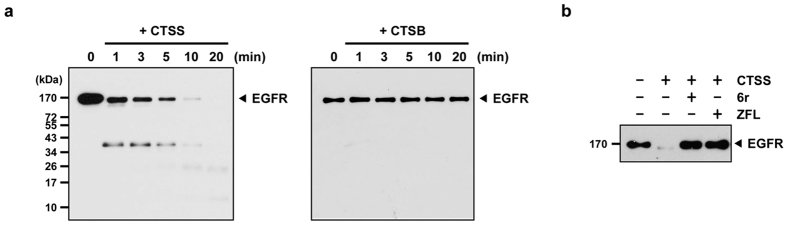
Proteolytic cleavage of EGFR by CTSS. (**a**) Recombinant EGFR was incubated with CTSS (left panel) and CTSB (right panel) at 37 °C for the indicated durations. The reaction was stopped by adding a sample buffer, and the reaction mixtures were subjected to SDS-PAGE, followed by Western blotting with an EGFR antibody. (**b**) Recombinant EGFR was incubated with CTSS in the presence of a CTSS inhibitor 6r or ZFL. The reaction mixtures were incubated at 37 °C for 20 min and then subjected to SDS-PAGE.

We previously observed that the inhibition of CTSS proteolytic activities by 6r or ZFL caused the activation of EGFR and its downstream signalling pathway, implying that CTSS is involved in the activation or degradation of EGFRs^35^. Considering the importance of endocytic degradation of active EGFR and of the natural proteolytic activity of CTSS, determining whether CTSS is involved in EGFR degradation is imperative. First, recombinant CTSS was incubated with EGFR to investigate the proteolytic role of CTSS in EGFR degradation. As shown in Fig. 1a (left panel), CTSS effectively cleaved EGFR at a neutral pH. After 10 min of incubation, CTSS nearly completely degraded EGFR. Nevertheless, no EGFR proteolysis was detected when EGFR was incubated with lysosomal CTSB (Fig. 1a, right panel). Next, we determined whether EGFR was actually degraded by the proteolytic activities of CTSS. As shown in Fig. 1b, EGFR degradation can be effectively inhibited by 6r or ZFL. Altogether, these findings revealed that the proteolytic activities of CTSS but not CTSB can effectively degrade EGFR.

**Figure 2:**
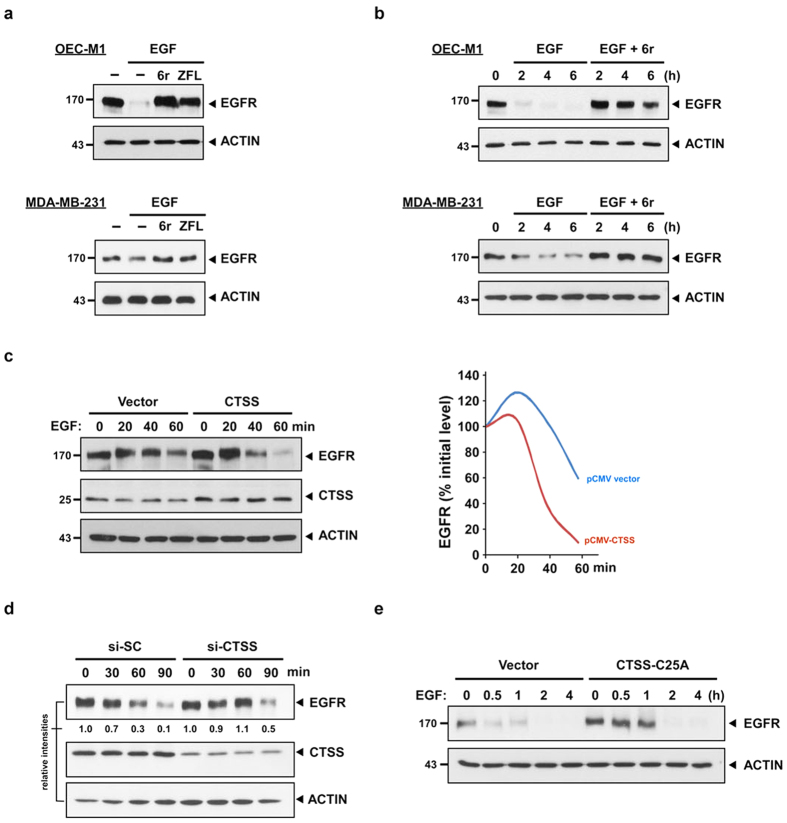
CTSS attenuates EGF-mediated EGFR degradation. (**a**) OEC-M1 and MDA-MB-231 cells were pretreated with 20 μM 6r or ZFL for 1 h and subsequently incubated with 100 ng/mL EGF for an additional 2 h. The total cell lysates were analysed using EGFR-specific antibodies. ACTIN was used as the internal control for semiquantitative loading in each lane. (**b**) The cells were stimulated with EGF (100 ng/mL) with or without the pretreatment of 20 μM 6r for the indicated durations. EGFR degradation was examined through immunostaining by using an anti-EGFR antibody. Notably, a substantial amount of EGFR was detectable even after 6 h of EGF stimulation in 6r-treated cells. (**c**) The OEC-M1 cells were transiently transfected with plasmids (pCMV) that encoded wild-type CTSS. After 24 h of transfection, the cells were treated with 100 ng/mL EGF for the indicated durations and the cellular EGFR, CTSS, and ACTIN signals were determined through Western blotting. The lifespan of EGF-mediated EGFR degradation was calculated by normalising the signal intensity of EGFR with that of ACTIN. (**d**) The MDA-MB-231 cells were transfected with specific 50 nM CTSS siRNA (si-CTSS) for 24 h and subsequently incubated with 100 ng/mL EGF for the indicated durations. The nontargeting scramble siRNA (si-SC) was used as the scramble control. (**e**) The MDA-MB-231 cells were transiently transfected with plasmids encoding the CTSS-C25A mutant. After 24 h of transfection, the cells were incubated with 100 ng/mL of EGF for the indicated durations. Furthermore, the cells were harvested and subjected to SDS-PAGE and Western blotting. EGFR degradation was determined using an antibody against EGFR. ACTIN signalling was included as the loading control.

In contrast to transforming growth factor-α and E4T, which each enhanced EGFR recycling, EGF stimulation was shown to promote EGFR degradation^36^. Therefore, the EGF ligand was subsequently used to examine whether CTSS activities were indeed involved in EGF-mediated EGFR degradation. After stimulation by EGF ligands for 1 h, EGF-mediated EGFR degradation was effectively observed in OEC-M1 and MDA-MB-231 cells; both cell types expressed wild-type EGFR (Fig. 2a). However, EGF-induced EGFR degradation was effectively reduced when the cells were pretreated with 6r or ZFL. Moreover, the delayed effect of EGF-mediated EGFR degradation was extended even after 6 h of EGF stimulation in CTSS-inhibited cells (Fig. 2b). To confirm whether CTSS was involved in EGFR degradation, the EGF-mediated EGFR degradation rates were determined in vector- and CTSS-overexpressing cells. As indicated in Fig. 2c, the lifespan of EGF-mediated EGFR degradation was reduced in CTSS-overexpressing cells. In addition, the siRNA-silencing approach was used to confirm the involvement of CTSS in EGF-mediated EGFR degradation. As shown in Fig. 2d, the EGFR degradation level was reduced in CTSS siRNA-transfected cells compared with that in scramble oligo-transfected control cells after 1 h of EGF treatment. Furthermore, the association between CTSS activities and EGFR degradation was validated using the CTSS inactive mutant CTSS-C25A. Site-directed mutagenesis was performed to change the cysteine to alanine in the active-site cleft of CTSS (Fig. S1a). Overexpression of the CTSS-C25A mutant can compete with the activities of endogenous wild-type CTSS, resulting in reduced total cellular CTSS activities (Fig. S1b). We then examined whether the competitive CTSS-C25A mutant affects the EGFR degradation process. Consistent with our aforementioned results, EGFR was barely detectable in vector control cells after 1 h of EGF treatment, whereas EGFR degradation was delayed in CTSS-C25A transfected cells (Fig. 2e). The delayed EGFR degradation may result from the remaining endogenous CTSS activities in cells with CTSS-C25A overexpression. To exclude the possibility that the delayed EGFR degradation in CTSS-inhibited cells was merely restricted to OEC-M1 and MDA-MB-231 cells, several human cancer cell lines, including human lung adenocarcinoma A549, human non-small cell lung carcinoma PC9, human pancreatic carcinoma Panc1, and human colon adenocarcinoma HT29, were examined. Western blotting revealed that the inhibition of CTSS activities in various cell lines delayed the EGF-mediated EGFR degradation process (Fig. S2). Collectively, these findings strongly suggest that CTSS plays a crucial role in the EGFR degradation process.

### CTSS inhibition prolongs EGFR occupancy in late endosomes

**Figure 3:**
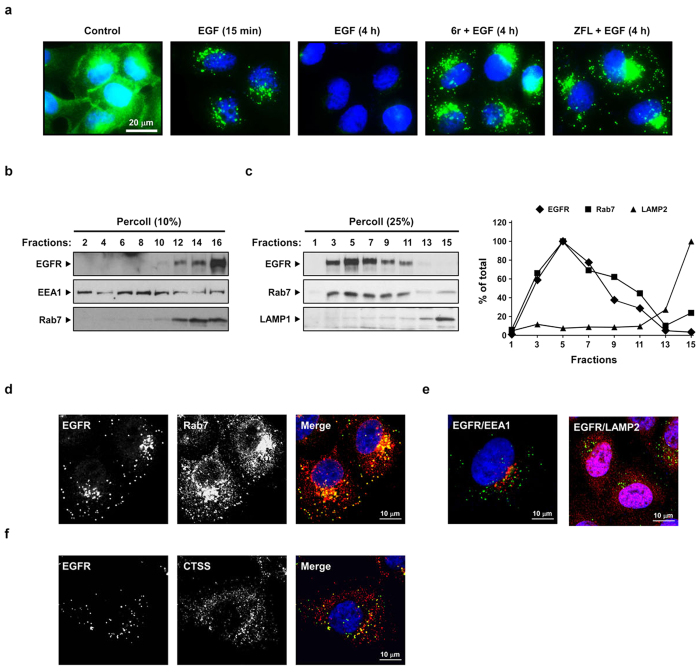
CTSS inhibition causes EGFR accumulation in late endosomes. (**a**) After the pretreatment of 20 μM 6r or ZFL for 1 h, the cells were stimulated with 100 ng/mL of EGF for the indicated durations. The cells were fixed, permeabilised, and immunostained for EGFR (green), as described in Methods. Nuclei were stained with DAPI. Note that CTSS inhibition caused EGFR accumulation in punctate intracellular vesicles. (**b,c**) The cells were pretreated with 20 μM 6r for 1 h and subsequently incubated with 100 ng/mL EGF for 1 h further. The cells were then homogenised and fractionated into gradients by using 10% Percoll (**b**) and 25% Percoll (**c**), as described in Methods. The Percoll gradient fractions were then subjected to SDS-PAGE, followed by Western blotting with EGFR, EEA1, Rab7, and LAMP1 antibodies. (**d–f**) The cells were pretreated with 20 μM 6r for 1 h and subsequently incubated with 100 ng/mL EGF for 2 h further and fixed, permeabilised, and stained with antibodies to EGFR (green; **d–f**) and Rab7 (red; d), EEA1 (red, e), LAMP2 (red, e), or CTSS (red; f). The cells were imaged through confocal microscopy. Scale bars, 10 μm. (**d**) Confocal images showed the colocalisation between accumulated EGFR and Rab7. (**e**) Confocal images showed that a very small amount of accumulated EGFR was colocalised with EEA1 or LAMP2. (**f**) Confocal images showing colocalisation between CTSS and accumulated EGFR.

The aforementioned findings suggest that CTSS activities participate in the process of EGFR endocytosis, vesicle trafficking, or EGFR degradation. We determined the subcellular location of EGFR after EGF stimulation in control or CTSS-inhibited cells. In the control cells, EGFRs were localised at the cell periphery (Fig. 3a, panel 1). After EGF stimulation for 15 min, intracellular EGFR punctate vesicles were predominantly stained within the cytosol, with almost no staining on cell surface (Fig. 3a, panel 2). Following 4 h of EGF stimulation, the EGFR-containing vesicles mostly disappeared (Fig. 3a, panel 3). By contrast, CTSS inhibition resulted in an enhanced accumulation of EGFR punctate vesicles in the perinuclear region (Fig. 3a, panels 4 and 5).

Next, we determined the intracellular location of accumulated EGFRs and examined whether inactive CTSS colocalised with these EGFRs. To address these concerns, CTSS-inhibited cells were treated with EGF for 2 h and were subsequently resolved in a 10% Percoll gradient to separate early and late endosomes. EEA1 and Rab7 were used as controls. As shown in Fig. 3b, accumulated EGFRs were predominantly distributed in the late endosomal fractions. To further validate whether the nondegraded EGFRs were predominantly located in late endosomal fractions, 25% Percoll gradient centrifugation was performed. Consistently, the EGFR signal was predominately distributed in fractions that corresponded to the late endosomes, but not to lysosomes, as indicated by LAMP2 (Fig. 3c). Furthermore, confocal microscopy supported the results that the accumulation of nondegraded EGFRs were largely colocalised with Rab7, rather than EEA1 or LAMP2 (Fig. 3d,e). We next examined whether the accumulated EGFR vesicles enclosed CTSS signals. As shown in Fig. 3f, the accumulated EGFR signals were colocalised with CTSS. Altogether, these results indicated that the inhibition of CTSS activities prolonged EGFR occupancy in the late endosomes.

### CTSS inhibition does not impair lysosomal activities

**Figure 4:**
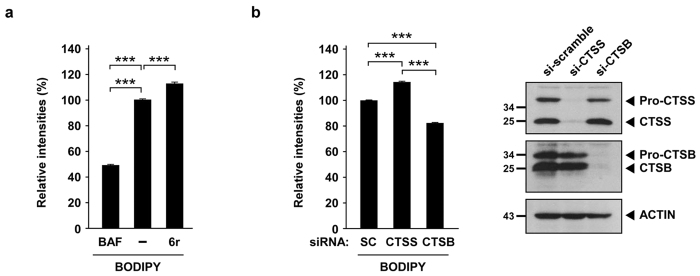
CTSS inhibition does not impair lysosomal activities. (**a**) OEC-M1 cells were pretreated with vesicle or 100 nM BAF for 1 h and then incubated with 20 μM 6r for 1 h further. Lysosomal proteolytic activities were determined using BODIPY–BSA and quantified through flow cytometry. (**b**) After 48 h of siRNA knockdown of CTSS and CTSB, the relative expression of CTSS and CTSB were determined through Western blotting (right panel). Lysosomal proteolytic activities were determined using BODIPY–BSA and quantified through flow cytometry (right panel). Data represent the mean ± SD of three independent experiments. Differences were found to be statistically significant at ***P < 0.001.

CTSS has been considered proteolytically active in lysosomes; therefore, it is imperative to examine whether CTSS inhibition impairs lysosomal proteolytic abilities. BODIPY–BSA was used to quantify lysosomal proteolytic abilities. As shown in Fig. 4a, the inhibition of lysosomal acidification by bafilomycin A1 (BAF) effectively impaired lysosomal proteolytic abilities, evidenced by the reduced fluorescence intensity of BODIPY–BSA. However, the pharmacological inhibition of CTSS activities by 6r further increased lysosomal proteolytic activities. Consistently, CTSS knockdown caused an increase in the fluorescence intensity of BODIPY–BSA, whereas lysosomal CTSB knockdown impaired lysosomal activities, as evidenced by a decreased fluorescence intensity of BODIPY–BSA (Fig. 4b). Altogether, these findings indicate that CTSS inhibition does not impair lysosomal proteolytic activities.

### Autophagy partially clears accumulated EGFR in CTSS-inhibited cells

**Figure 5:**
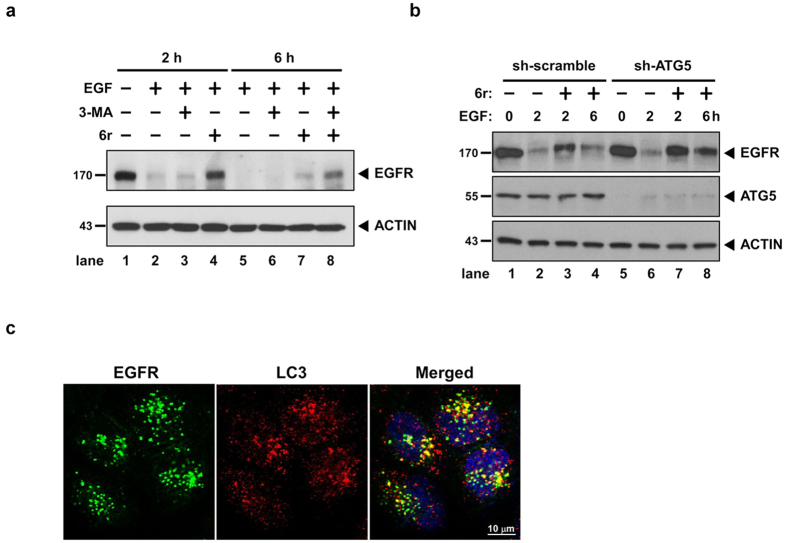
Autophagy is involved in the clearance of accumulated EGFR. (**a**) The cells were pretreated with 10 mM 3-MA or 20 μM 6r for 1 h and then stimulated with 100 ng/mL EGF for additional periods of 2 and 6 h. Subsequently, the cells were harvested and subjected to SDS-PAGE and Western blotting. EGFR degradation was examined through immunostaining by using an anti-EGFR antibody. ACTIN signalling was considered the loading control. (**b**) After 24 h of shRNA knockdown of CTSS, the cells were treated with 100 ng/mL EGF for the indicated durations. The harvested cells were subjected to Western blotting. (**c**) The cells were pretreated with 20 μM 6r for 1 h and then treated with 100 ng/mL EGF for 30 min. The cells were immediately fixed, permeabilised, stained with EGFR and LC3 antibodies, as described in Methods.

Our previous studies have indicated that CTSS inhibition induces autophagy activation through the EGFR signalling pathway^30^,^31^. However, the relationship among autophagy activation, EGFR signalling, and CTSS activities remain unclear. Therefore, we next examined whether autophagy activation is crucial for clearing EGFR accumulation in CTSS-inhibited cells. As shown in Fig. 5a, basal autophagy is slightly involved in EGFR degradation because the pharmacological inhibition of autophagy by 3-MA only slightly attenuates EGF-mediated EGFR degradation (compare lanes 2 and 3). Consistently, the EGFR degradation process was delayed in the CTSS-inhibited cells after 2 h of EGF treatment, and the EGFR amount gradually decreased after 6 h of EGF treatment (Fig. 5a, compare lanes 4 and 7). Notably, EGFR degradation was further attenuated when autophagy was inhibited by 3-MA (Fig. 5a, compare lanes 7 and 8). Moreover, the small hairpin (sh) RNA approach was used to knockdown autophagy-related protein 5 (ATG5) proteins that are necessary for autophagy activation. In accordance with the 3-MA treatment, ATG5 knockdown slightly attenuated EGF-mediated EGFR degradation (Fig. 5b, compare lanes 2 and 6). However, under 6r-treated conditions, ATG5 knockdown resulted in increased EGFR accumulation (Fig. 5b, compare lanes 3 and 7 as well as lanes 4 and 8). To confirm whether autophagy plays a crucial role in the clearance of accumulated EGFR, we performed confocal analysis to examine the colocalisation of accumulated EGFR and LC3, which is a critical marker of autophagy. As shown in Fig. 5c, our results indicated that EGFR-positive vesicles were colocalised with LC3 when 6r-treated cells were treated with EGF for 30 min, suggesting that autophagy is involved in EGFR degradation in the early stage. Collectively, findings indicate that autophagy at least partially promotes the clearance of accumulated EGFR in CTSS-inhibited cells.

### CTSS inhibition results in a prolonged EGFR activation and sustained AKT and STAT3 activation

**Figure 6:**
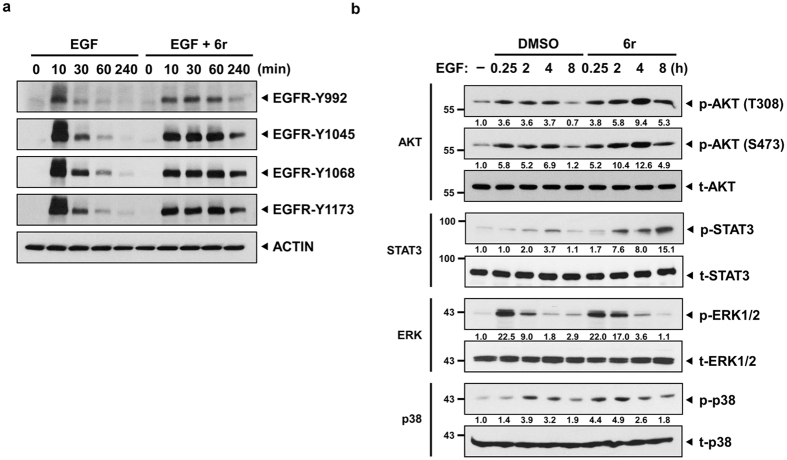
CTSS inhibition results in a prolonged EGFR activation and sustained downstream signaling. (**a**) OEC-M1 cells with and without the treatment of 20 μM 6r were stimulated with 100 ng/mL EGF for the indicated durations. Cell lysates were separated through SDS-PAGE and then probed with the indicated phospho-EGFR antibodies. (**b**) After 1 h of pretreatment of 20 μM 6r, the cells were stimulated with 100 ng/mL EGF for the indicated durations. The harvested cells were then subjected to Western blotting analysis and probed with the indicated antibodies. The relative band intensities were shown.

Following EGF ligand binding, the EGFR undergoes a transition from an inactive monomeric form to an active dimer form and then stimulates the autophosphorylation of tyrosine (Y) residues in the C-terminal domain of the EGFR. Thus, we subsequently assessed whether the EGFR signalling cascades were prolonged in CTSS-inhibited cells. In the control cells, the EGF treatment immediately increased EGFR-Y992, -Y1045, -Y1068, and -Y1173 phosphorylation within 10 min, and the phosphorylation levels gradually declined to baseline after 240 min of EGF treatment (Fig. 6a). However, in 6r-treated cells, the degradation of phosphorylated EGFR decreased and resulted in sustained EGFR activation even after 240 min of EGF treatment. We next investigated whether the sustained EGFR activation in 6r-treated cells could further prolong EGFR downstream signalling. As shown in Fig. 6b, EGF triggered an increase in the phosphorylation of STAT3, AKT, ERK1/2, and p38 as early as 15 min and sustained for 4 h (except p-ERK1/2); the phosphorylation started declining 8 h after the treatment. However, the phosphorylation intensity of these signals was stronger in the cells cotreated with 6r and EGF than in the cells treated with EGF alone.

### Enhancement of cell apoptosis with the combination of 6r and Iressa

**Figure 7:**
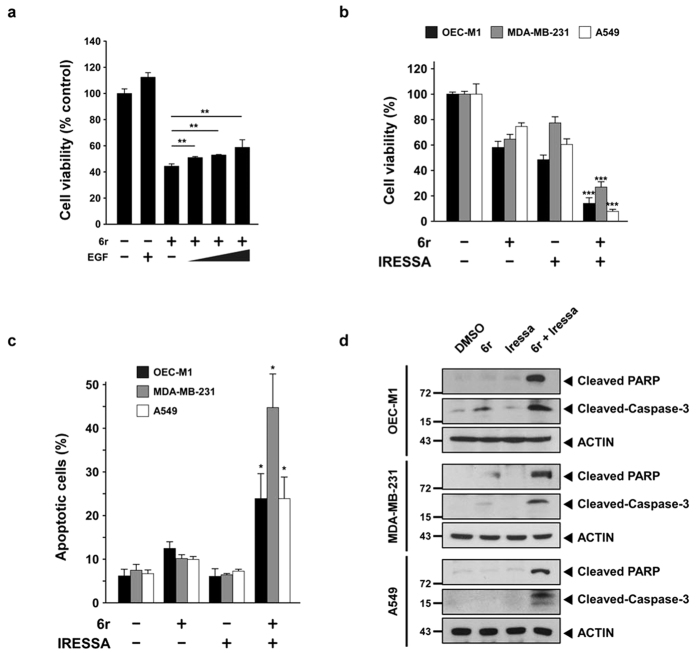
Cotreatment with 6r and Iressa enhances cancer cells apoptosis. (**a**) OEC-M1 cells were pretreated with or without 20 μM 6r for 1 h and subsequently incubated with 100 ng/mL EGF for an additional 72 h. Cell viabilities were determined by a methylene blue dye assay. The bars represent the relative mean survival of four independent wells ± SD. Differences were considered statistically significant at *P < 0.05, **P < 0.01, and ***P < 0.001. (**b**) The cells were treated with 6r alone or in combination with Iressa for 72 h. Cell viabilities were determined by a methylene blue dye assay. In the OEC-M1 cells, 20 μM 6r and 10 μM Iressa were used. For the MDA-MB-231 cells, 15 μM 6r and 15 μM Iressa were used. Moreover, 40 μM 6r and 27 μM Iressa were used in the A549 cells. (**c**) After 24 h of treatment with 6r or Iressa or the 6r–Iressa combination, cell apoptotic rates were evaluated with annexin V/propidium iodide double staining. The data represent the mean ± SD of three independent experiments. Differences were considered statistically significant when *P < 0.05, **P < 0.01, and ***P < 0.001. (**d**) The cells were treated with 6r, Iressa, or the 6r–Iressa combination for 24 h; cleaved PARP and caspase-3 were assessed through Western blotting.

We then investigated whether prolonged EGFR signalling could induce cell cytotoxicity or lead to cell survival. The EGF treatment significantly (*P* < 0.005) increased the cell viability not only in the vehicle-treated but also in the CTSS-inhibited cells (Fig. 7a). We next evaluated whether the combination of 6r and Iressa exerted stronger cytotoxic effects on various tumour cell lines. The cells were treated with 6r or Iressa alone or in concomitant combination at a fixed molar ratio. Notably, the combination of 6r and Iressa reduced the cell viability of OEC-M1, MDA-MB-231, and A549 tumour cells (Fig. 7b). To determine whether the inhibitory effect of the combination was caused by enhanced tumour apoptosis, an annexin V-FITC apoptotic assay was used to determine the apoptotic rate. As shown in Fig. 7c, apoptosis of the cells treated with 6r alone slightly increased, whereas no apoptosis was observed for the cells treated with Iressa alone. The combination of 6r and Iressa markedly increased the percentage of apoptotic cells. To confirm whether the 6r and Iressa cotreatment enhanced tumour apoptosis, apoptotic hallmarks were examined by detecting the amount of cleaved caspase-3 and polyadenosine diphosphate ribose polymerase (PARP). As shown in Fig. 7d, a noticeable amount of cleaved caspase-3 and cleaved PARP signals were observed in the cells cotreated with 6r and Iressa than those treated with 6r or Iressa alone. Collectively, these experiments indicated that the combination of 6r and Iressa markedly enhanced apoptosis in the OEC-M1, MDA-MB-231, and A549 cells.

## Discussion

Ligand-stimulated EGFR can activate the signalling cascade from the plasma membrane^4^, recruit signalling molecules, and uninterruptedly transmit signals from endocytic endosomes^5^,^37^. Thus, spatial and temporal compartmentalisation of the activated EGFR among the plasma membrane, early endosomes, and late endosomes can regulate the amplitude, timing, and specificity of signalling cascades. EGFR endocytosis from the plasma membrane to early endosomes is required for the full spectrum of EGF-mediated ERK1/2 and PI3K/AKT signalling^38^. In addition, STAT3 signalling was reported to interact with the MAPK signalling system at late endosomal structures^39^. Considering the spatial regulation of late endosomes, Taub *et al*. observed that the mislocalisation of late endosomal EGFR could transmit different signalling cascades; the accumulation of late endosomes in the cell peripheral region prolonged ERK and p38 signalling, whereas the clustering of late endosomes in the perinuclear region only resulted in the sustained phosphorylation of ERK^5^. The present study revealed EGFR accumulation in the perinuclear region after EGF treatment in CTSS-inhibited cells (Fig. 3a). However, no difference was observed in the half-life of the phosphorylated ERK level between the control and CTSS-inhibited cells (Fig. 6b). By contrast, only sustained AKT and STAT3 signalling was observed. The difference was likely caused by different EGFR degradation kinetics and various biological roles in EGFR signalling regulation. In the dominant active Rab7 expression model (Rab7da) developed by Taub, Rab7da overexpression delayed the entry of the EGFR into the late endosomes, causing a delay in EGFR degradation and sustaining pERK1/2 signalling^5^. However, EGFR degradation in the Rab7da model was only delayed up to 1 h. This indicated that although Rab7da overexpression mislocalised late endosomes in the perinuclear region, fewer EGFRs were exhibited in these late endosomes after 2 h of EGF stimulation. By contrast, CTSS inhibition not only reduced EGFR degradation but also caused EGFR accumulation in late endosomes at the perinuclear region even after 4 h EGF stimulation (Fig. 2b). Furthermore, the accumulation of the activated EGFR in late endosomes could uninterruptedly transmit signalling, thus prolonging STAT3 and AKT signalling.

Autophagy is typically sustained in a low basal level for cellular homeostasis. Under stress, autophagy is upregulated to eliminate damaged molecules, misfolded proteins, and organelles to compensate for environmental stress. Autophagic and endocytic processes eventually congregate into lysosomes to acquire active proteases for cargo degradation. Therefore, autophagosomes have been reported to fuse with damaged endosomes for autophagic clearance^40^,^41^. Notably, our previous study revealed that CTSS inhibition triggers autophagy activation^30^. Furthermore, our current data revealed that CTSS inhibition prolongs EGFR occupancy in late endosomes (Fig. 3). Therefore, it can be speculated that autophagy may be responsible for autophagic clearance of accumulated EGFRs in late endosomes. Our findings showed that early endocytic EGFR puncta largely colocalised with LC3 in CTSS-inhibited cells within 30 min of EGF treatment (Fig. 5c). Razi also reported that autophagosomes could fuse with early endosomes during an autophagic flux^42^. However, autophagy inhibition can only partially rescue EGF-mediated EGFR degradation in cells cotreated with a CTSS inhibitor (Fig. 5a,b). Furthermore, most of these accumulated EGFR-containing vesicles did not show LC3-labelling signals in CTSS-inhibited cells after 2 h of EGF treatment. These results implied that autophagy only participates in the clearance of endocytic EGFR vesicles during the early phase of EGF stimulation but not in the elimination of accumulated EGFR vesicles during the late endosomal stage. In addition, functional multivesicular bodies were reported to be required for efficient autophagic degradation^40^. In this study, the EGFR-containing vesicles were highly associated with CTSS-labelling signals (Fig. 3f), but not with LC3-labelling signals in CTSS-inhibited cells after 2 h of EGF treatment. Thus, CTSS is likely to play a pivotal role in the functional regulation of multivesicular bodies. CTSS inhibition may impair the maturation of multivesicular bodies, thus weakening the autophagic clearance ability of accumulated late endosomes or multivesicular bodies; this warrants future study.

Lysosomes, membrane-bound vesicles, carry a bulk of mature acid hydrolases to fuse late endosomes or autophagosomes for enzymatic degradation. Thus, lysosomes are considered the final destination of degradative compartments in the cells. Lysosomes maintain the acidic pH gradient in the range of 4–5^43^ by pumping protons into the lysosome lumen via proton-pumping vacuolar-type ATPase^44^,^45^, thus providing an optimal environment for acid hydrolases such as CTSB^46^. The inhibition of lysosomal acidification by BAF or attenuation of lysosomal CTSB activities^47^ impair the lysosomal function and subsequent protein degradation. CTSS was typically considered a lysosomal cathepsin because CLARK *et al*. observed that CTSS staining was partially colocalised with LAMP1^15^ when cells were treated with LPS and ATP. However, unlike CTSB, CTSS inhibition slightly increased lysosomal activities, as evidenced by an increased fluorescence intensity of lysotracker red and by elevated CTSB activities^31^. It remains unclear whether CTSS inhibition impairs the lysosomal proteolytic activity, which subsequently leads to EGFR accumulation. In the present study, in contrast to CTSB silencing-reduced lysosomal proteolytic activities, an increased lysosomal degradation of BODIPY–BSA was observed in both CTSS-inhibited and CTSS-silenced cells (Fig. 4). These findings suggest that CTSS is not a crucial member of lysosomal hydrolases for lysosomal degradation but is proteolytically active in the early or late endosomes before sorting into lysosomes.

In conclusion, the present findings reveal that CTSS plays a crucial role in EGFR signalling attenuation before sorting into lysosomes. CTSS inhibition decelerated the EGFR degradation process and caused EGFR accumulation in late endosomes. Crucially, a combination of 6r and Iressa markedly promoted tumour apoptosis. Altogether, our study not only identified a new biological role of CTSS in EGFR signalling regulation, but also provided a mechanical rationale to combine a CTSS inhibitor and an EGFR tyrosine kinase inhibitor for treating cancer through EGFR expression in the future.

## Methods

### Chemicals

The specific CTSS inhibitor 6r was synthesised and provided by Professor Chun-Cheng Lin of the Department of Chemistry, National Tsing Hua University, Hsinchu, Taiwan. The commercial CTSS inhibitor Z-FL-COCHO (ZFL, #219393) was purchased from Calbiochem. Moreover, the recombinant EGFR (#2645), 3-methyladenine (3-MA, #M9281), bafilomycin A1 (#B1793), and MES (#M5057) were all purchased from Sigma. EGF (AF-100-15) was purchased from PeproTech. A self-quenched BODIPY FL conjugate of BSA (green, #7932) was purchased from BioVision.

### Cell culture

Human oral squamous cell carcinoma (OEC-M1) and human breast adenocarcinoma (MDA-MB-231) cells were grown in an RPMI-1640 medium (Gibco) supplemented with 10% foetal bovine serum (FBS), 2 mM glutamine, 100 U/mL penicillin, and 100 μg/mL streptomycin at 37 °C in an atmosphere of 95% air and 5% CO2. A549 human lung adenocarcinoma cells were maintained in Kaighn’s modification of Ham’s F12 medium (Gibco) supplemented with 2 mM glutamine, 100 U/mL penicillin, and 100 μg/mL streptomycin, and 10% FBS.

### Antibodies

CTSS (#AF1183) and CTSB (#AF953) antibodies were purchased from R&D Systems. Anti-actin (#MAB1501) and anti-STAT3 (#610190) antibodies were purchased from Millipore and BD Transduction Laboratories, respectively. Antibodies against EGFR (#sc-03 and #sc-377073), EGFR-phospho Y1173 (#sc-12351), and LAMP1 (#5570) were obtained from Santa Cruz. A phospho-p38 (pThr180/182) antibody (#44-684G) was obtained from Invitrogen. An antibody against Rab7 (#ab137029) was purchased from Abcam. Furthermore, antibodies against EGFR-phospho Y992 (#2235), EGFR-phospho Y1045 (#2237), EGFR-phospho Y1068 (#2234), Rab7 (#9367), phospho-STAT3 (#9134), total p38 (#9212), total ERK (#9102), phospho-ERK1/2 (#9101), AKT-T308 (#2965), AKT-S473 (#9271), EEA1 (#3288), and Rab7 (#9367) were purchased from Cell Signaling Technology.

### EGFR hydrolysis by CTSS

Recombinant EGFR (600 ng) was incubated with 5 ng CTSS or cathepsin B (CTSB) in a reaction buffer [100 mM MES (pH 6.0), 16 mM dithiothreitol, and 1.6 mM ethylenediaminetetraacetic acid (EDTA)] at 37 °C for 0–20 min. To inhibit cathepsin activities, the same reaction mixtures were prepared but with the addition of 6r^31^ or ZFL, which are inhibitors of CTSS. The reacted samples were separated through sodium dodecyl sulfate polyacrylamide gel electrophoresis (SDS-PAGE) and subsequently transferred onto polyvinylidene fluoride (PVDF) membranes for EGFR immunodetection. The immunoblots were blocked with 5% nonfat milk for 1 h and subsequently blotted with the indicated specific primary antibodies against CTSS or CTSB. The blotted membranes were washed with Tris-buffered saline (TBS) and TBST (TBS containing 0.1% Tween 20) and blotted with horseradish peroxidase-conjugated secondary antibodies. Finally, the chemiluminescent signal of EGFR was visualised by exposure to X-ray for the appropriate duration by using the Western Lightning Plus ECL system (Perkin Elmer).

### Gene silencing by siRNA oligonucleotides

The specific siRNA against CTSB (#sc-29238) and scramble control siRNA (#sc-37007) were purchased from Santa Cruz. Synthetic double-strand siRNA oligonucleotides against CTSS were purchased from Invitrogen. The target sequence of CTSS is 5′-CCACAACUUUGGUGAAGAA-3′. Gene silencing was performed using an RNAiMax reagent (Invitrogen) by following the manufacturer’s instructions. In brief, the cells were seeded onto six-well culture plates and allowed to grow overnight to reach an appropriate confluence. The siRNA oligonucleotides and 7.5 μL of the RNAiMAX reagent were separately diluted with 100 μL of Opti-MEM medium (Invitrogen) and then mixed. After 20 min of incubation at room temperature, the cells were incubated with the aforementioned well-optimised transfection medium and incubated for 24 h at 37 °C in an atmosphere of 95% air and 5% CO2.

### Percoll gradient fractionation

The cells were pretreated with 20 μM 6r for 1 h and subsequently incubated with 100 ng/mL EGF for 1 h further. After washing twice with phosphate-buffered saline (PBS), the cells were scraped off with 2 mL lysis buffer (10 mM ethanolamine, 1 mM EDTA, and 0.25 M sucrose, pH 7.2) and centrifuged at 200 × g for 10 min. The supernatant was then transferred into a new tube, and the pellet was resuspended with 2 mL homogenisation buffer. After additional centrifugation at 200 × g for 10 min, the supernatant was pooled, yielding 4 mL of postnuclear supernatant. Subsequently, the postnuclear supernatant was diluted with a 90% Percoll reagent (#17-0891-02; GE Healthcare) to a final concentration of 8 mL 10% or 25% Percoll. Percoll-containing samples were centrifuged for 25 min at 4 °C at 50 000 × g in a Beckman Ti70.1 rotor. Finally, the gradients were fractionated into 16 fractions from the top to the bottom. The fractions were then immunoblotted for an EGFR, an early endosomal marker (EEA1), a late endosomal marker (Rab7), and a lysosomal marker (LAMP2), as indicated in the figure.

### Quantification of lysosomal function and proteolytic activity

BODIPY–BSA was used to determine the lysosomal functions and proteolytic activities of CTSS or CTSB, according to the manufacturer’s instructions. In brief, OEC-M1 cells were seeded into a 6-cm culture dish and allowed to grow overnight to yield approximately 80% confluence. The cells were pretreated with dimethyl sulfoxide or 20 μM 6r for 1 h and subsequently incubated with BODIPY–BSA (final concentration, 10 μg/mL) for 3 h. The cells were then harvested, and the fluorescence intensity of BODIPY–BSA were quantified using a FACSVantage flow cytometric analyser (BD FACSCalibur) and analysed using CellQuest software. For each assay, 10000 events were analysed. Our results are presented as the mean ± standard deviation (SD) of three independent experiments.

### Immunofluorescence analysis

Immunofluorescent staining was performed as previously described^32^. In brief, the cells grown on CultureSlides (BD Biosciences) were exposed to the indicated reagents, fixed with 4% paraformaldehyde, washed twice with ice-cold PBS, permeabilised with 0.1% Triton X-100, and then blocked with 1% BSA for 1 h at room temperature. Subsequently, the cells were incubated with the indicated primary antibody for 16 h at 4 °C. After washing with PBS to remove unbound primary antibodies, bound antibodies were visualised by incubating the cells with secondary antibodies (Molecular probes). 4′,6-Diamidino-2-phenylindole (DAPI) was used as a counterstain to visualise the nuclei. The fluorescent images were obtained using a Leica DMI 4000B microscope with appropriate filters and lasers. For confocal analysis, the images were obtained using a FV1000 confocal microscope (Olympus) with a 60 × oil immersion lens, NA 1.35 (Uplsapo).

### SDS-PAGE and Western blotting

Samples for analysis were resuspended in a lysis buffer (#C2978; Sigma) containing 1 mM Na3VO4, 0.5 mM PMSF, and 1 × protease inhibitor cocktail. After being sonicated and centrifuged at 14000 rpm for 10 min to remove debris, the protein concentration was analysed using a BCA protein assay kit (#23225; Pierce). An equal amount of proteins was resuspended in a 1 × SDS-PAGE sample buffer (2% SDS, 12.5 mM EDTA, 1% β-mercaptoethanol, 20% v/v glycerol, 0.02% bromophenol blue, and 50 mM Tris-Cl, pH 6.8) in a boiling water bath for 10 min and subsequently subjected to electrophoresis in polyacrylamide gels. Proteins were transferred onto PVDF membranes and blocked in TBS (150 mM NaCl and 10 mM Tris-Cl, pH 7.4) containing 5% w/v nonfat milk. The membranes were probed with specific primary antibodies and then treated using secondary antibodies conjugated with horseradish peroxidase (Santa Cruz). The chemiluminescent signal was visualised using a Western Lightning Plus ECL reagent (Perkin Elmer) with an appropriate time of exposure to Kodak Biomax films.

### Statistical analysis

In this study, all analysed data are presented as the mean ± SD. Differences were evaluated using the unpaired two-tailed Student *t* test. The *P* values are denoted with asterisks: *P < 0.05, **P < 0.01, and ***P < 0.001; In this study, *P* < 0.05 was considered statistically significant.

## Additional Information

**How to cite this article**: Huang, C.-C. *et al*. Cathepsin S attenuates endosomal EGFR signalling: A mechanical rationale for the combination of cathepsin S and EGFR tyrosine kinase inhibitors. *Sci. Rep.* **6**, 29256; doi: 10.1038/srep29256 (2016).

## Supplementary Material

Supplementary Information

## References

[b1] Saranath D. . Amplification and overexpression of epidermal growth factor receptor gene in human oropharyngeal cancer. Eur J Cancer B Oral Oncol 28B, 139–143 (1992). 1306731 10.1016/0964-1955(92)90043-z

[b2] Rush J. S., Quinalty L. M., Engelman L., Sherry D. M. & Ceresa B. P. Endosomal accumulation of the activated epidermal growth factor receptor (EGFR) induces apoptosis. J Biol Chem 287, 712–722 (2012). 22102283 10.1074/jbc.M111.294470 PMC3249126

[b3] Hyatt D. C. & Ceresa B. P. Cellular localization of the activated EGFR determines its effect on cell growth in MDA-MB-468 cells. Exp Cell Res 314, 3415–3425 (2008). 18817771 10.1016/j.yexcr.2008.08.020 PMC2590673

[b4] Sousa L. P. . Suppression of EGFR endocytosis by dynamin depletion reveals that EGFR signaling occurs primarily at the plasma membrane. Proc Natl Acad Sci USA 109, 4419–4424 (2012). 22371560 10.1073/pnas.1200164109 PMC3311323

[b5] Taub N., Teis D., Ebner H. L., Hess M. W. & Huber L. A. Late endosomal traffic of the epidermal growth factor receptor ensures spatial and temporal fidelity of mitogen-activated protein kinase signaling. Mol Biol Cell 18, 4698–4710 (2007). 17881733 10.1091/mbc.E07-02-0098 PMC2096590

[b6] Pennock S. & Wang Z. Stimulation of cell proliferation by endosomal epidermal growth factor receptor as revealed through two distinct phases of signaling. Mol Cell Biol 23, 5803–5815 (2003). 12897150 10.1128/MCB.23.16.5803-5815.2003 PMC166318

[b7] Sadowski L., Pilecka I. & Miaczynska M. Signaling from endosomes: location makes a difference. Exp Cell Res 315, 1601–1609 (2009). 18930045 10.1016/j.yexcr.2008.09.021

[b8] Tjelle T. E., Brech A., Juvet L. K., Griffiths G. & Berg T. Isolation and characterization of early endosomes, late endosomes and terminal lysosomes: their role in protein degradation. J Cell Sci 109 (Pt 12), 2905–2914 (1996). 9013338 10.1242/jcs.109.12.2905

[b9] Kirschke H. & Wiederanders B. Cathepsin S and related lysosomal endopeptidases. Methods Enzymol 244, 500–511 (1994). 7845228 10.1016/0076-6879(94)44036-0

[b10] Bromme D. . Functional expression of human cathepsin S in Saccharomyces cerevisiae. Purification and characterization of the recombinant enzyme. J Biol Chem 268, 4832–4838 (1993). 8444861

[b11] McGrath M. E., Palmer J. T., Bromme D. & Somoza J. R. Crystal structure of human cathepsin S. Protein Sci 7, 1294–1302 (1998). 9655332 10.1002/pro.5560070604 PMC2144034

[b12] Turkenburg J. P. . Structure of a Cys25– >Ser mutant of human cathepsin S. Acta Crystallogr D Biol Crystallogr 58, 451–455 (2002). 11856830 10.1107/s0907444901021825

[b13] Freier R., Dall E. & Brandstetter H. Protease recognition sites in Bet v 1a are cryptic, explaining its slow processing relevant to its allergenicity. Sci Rep 5, 12707 (2015). 26235974 10.1038/srep12707 PMC4522599

[b14] Lindahl C. . Increased levels of macrophage-secreted cathepsin S during prostate cancer progression in TRAMP mice and patients. Cancer Genomics Proteomics 6, 149–159 (2009). 19487544

[b15] Clark A. K., Wodarski R., Guida F., Sasso O. & Malcangio M. Cathepsin S release from primary cultured microglia is regulated by the P2X7 receptor. Glia 58, 1710–1726 (2010). 20629190 10.1002/glia.21042

[b16] Fernandez P. L. . Expression of cathepsins B and S in the progression of prostate carcinoma. Int J Cancer 95, 51–55 (2001). 11241311 10.1002/1097-0215(20010120)95:1<51::aid-ijc1009>3.0.co;2-j

[b17] Gormley J. A. . The role of Cathepsin S as a marker of prognosis and predictor of chemotherapy benefit in adjuvant CRC: a pilot study. Br J Cancer 105, 1487–1494 (2011). 21989182 10.1038/bjc.2011.408 PMC3242524

[b18] Xu J. . Cathepsin S is aberrantly overexpressed in human hepatocellular carcinoma. Mol Med Rep 2, 713–718 (2009). 21475890 10.3892/mmr_00000161

[b19] Flannery T. . The clinical significance of cathepsin S expression in human astrocytomas. Am J Pathol 163, 175–182 (2003). 12819022 10.1016/S0002-9440(10)63641-3 PMC1868175

[b20] Flannery T. . Cathepsin S expression: An independent prognostic factor in glioblastoma tumours–A pilot study. Int J Cancer 119, 854–860 (2006). 16550604 10.1002/ijc.21911

[b21] Wang B. . Cathepsin S controls angiogenesis and tumor growth via matrix-derived angiogenic factors. J Biol Chem 281, 6020–6029 (2006). 16365041 10.1074/jbc.M509134200

[b22] Shi G. P. . Deficiency of the cysteine protease cathepsin S impairs microvessel growth. Circ Res 92, 493–500 (2003). 12600886 10.1161/01.RES.0000060485.20318.96

[b23] Fan Q. . Silencing cathepsin S gene expression inhibits growth, invasion and angiogenesis of human hepatocellular carcinoma *in vitro*. Biochem Biophys Res Commun 425, 703–710 (2012). 22796222 10.1016/j.bbrc.2012.07.013

[b24] Reddy V. Y., Zhang Q. Y. & Weiss S. J. Pericellular mobilization of the tissue-destructive cysteine proteinases, cathepsins B, L, and S, by human monocyte-derived macrophages. Proc Natl Acad Sci USA 92, 3849–3853 (1995). 7731994 10.1073/pnas.92.9.3849 PMC42059

[b25] Blondeau X. . Generation of matrix-degrading proteolytic system from fibronectin by cathepsins B, G, H and L. Biol Chem Hoppe Seyler 374, 651–656 (1993). 8240719 10.1515/bchm3.1993.374.7-12.651

[b26] Taleb S., Cancello R., Clement K. & Lacasa D. Cathepsin s promotes human preadipocyte differentiation: possible involvement of fibronectin degradation. Endocrinology 147, 4950–4959 (2006). 16825321 10.1210/en.2006-0386

[b27] Lee-Dutra A., Wiener D. K. & Sun S. Cathepsin S inhibitors: 2004–2010. Expert Opin Ther Pat 21, 311–337 (2011). 21342054 10.1517/13543776.2011.553800

[b28] Chen J. C. . Design and synthesis of alpha-ketoamides as cathepsin S inhibitors with potential applications against tumor invasion and angiogenesis. J Med Chem 53, 4545–4549 (2010). 20481438 10.1021/jm100089e

[b29] Small D. M. . Cathepsin S from both tumor and tumor-associated cells promote cancer growth and neovascularization. Int J Cancer 133, 2102–2112 (2012). 23629809 10.1002/ijc.28238

[b30] Chen K. L. . Targeting cathepsin S induces tumor cell autophagy via the EGFR-ERK signaling pathway. Cancer Lett 317, 89–98 (2012). 22101325 10.1016/j.canlet.2011.11.015

[b31] Huang C. C., Chen K. L., Cheung C. H. & Chang J. Y. Autophagy induced by cathepsin S inhibition induces early ROS production, oxidative DNA damage, and cell death via xanthine oxidase. Free Radic Biol Med 65, 1473–1486 (2013). 23892358 10.1016/j.freeradbiomed.2013.07.020

[b32] Huang C. C. . Autophagy-Regulated ROS from Xanthine Oxidase Acts as an Early Effector for Triggering Late Mitochondria-Dependent Apoptosis in Cathepsin S-Targeted Tumor Cells. PLoS One 10, e0128045 (2015). 26029922 10.1371/journal.pone.0128045 PMC4452096

[b33] Zhang L., Wang H., Xu J., Zhu J. & Ding K. Inhibition of cathepsin S induces autophagy and apoptosis in human glioblastoma cell lines through ROS-mediated PI3K/AKT/mTOR/p70S6K and JNK signaling pathways. Toxicol Lett 228, 248–259 (2014). 24875536 10.1016/j.toxlet.2014.05.015

[b34] Wang X. . Cathepsin S silencing induces apoptosis of human hepatocellular carcinoma cells. Am J Transl Res 7, 100–110 (2015). 25755832 PMC4346527

[b35] Chen K. L. . Targeting cathepsin S induces tumor cell autophagy via the EGFR-ERK signaling pathway. Cancer Lett 317, 89–98 (2012). 22101325 10.1016/j.canlet.2011.11.015

[b36] Alwan H. A., van Zoelen E. J. & van Leeuwen J. E. Ligand-induced lysosomal epidermal growth factor receptor (EGFR) degradation is preceded by proteasome-dependent EGFR de-ubiquitination. J Biol Chem 278, 35781–35790 (2003). 12829707 10.1074/jbc.M301326200

[b37] von Zastrow M. & Sorkin A. Signaling on the endocytic pathway. Curr Opin Cell Biol 19, 436–445 (2007). 17662591 10.1016/j.ceb.2007.04.021 PMC1992519

[b38] Vieira A. V., Lamaze C. & Schmid S. L. Control of EGF receptor signaling by clathrin-mediated endocytosis. Science 274, 2086–2089 (1996). 8953040 10.1126/science.274.5295.2086

[b39] German C. L., Sauer B. M. & Howe C. L. The STAT3 beacon: IL-6 recurrently activates STAT 3 from endosomal structures. Exp Cell Res 317, 1955–1969 (2011). 21619877 10.1016/j.yexcr.2011.05.009 PMC3788646

[b40] Filimonenko M. . Functional multivesicular bodies are required for autophagic clearance of protein aggregates associated with neurodegenerative disease. J Cell Biol 179, 485–500 (2007). 17984323 10.1083/jcb.200702115 PMC2064794

[b41] Chen X. . Autophagy induced by calcium phosphate precipitates targets damaged endosomes. J Biol Chem 289, 11162–11174 (2014). 24619419 10.1074/jbc.M113.531855 PMC4036255

[b42] Razi M., Chan E. Y. & Tooze S. A. Early endosomes and endosomal coatomer are required for autophagy. J Cell Biol 185, 305–321 (2009). 19364919 10.1083/jcb.200810098 PMC2700373

[b43] Poole B. & Ohkuma S. Effect of weak bases on the intralysosomal pH in mouse peritoneal macrophages. J Cell Biol 90, 665–669 (1981). 6169733 10.1083/jcb.90.3.665 PMC2111912

[b44] Bowman E. J., Siebers A. & Altendorf K. Bafilomycins: a class of inhibitors of membrane ATPases from microorganisms, animal cells, and plant cells. Proc Natl Acad Sci USA 85, 7972–7976 (1988). 2973058 10.1073/pnas.85.21.7972 PMC282335

[b45] Mindell J. A. Lysosomal acidification mechanisms. Annu Rev Physiol 74, 69–86 (2012). 22335796 10.1146/annurev-physiol-012110-142317

[b46] Choi S. Y. . Multiple cell death pathways are independently activated by lethal hypertonicity in renal epithelial cells. Am J Physiol Cell Physiol 305, C1011–C1020 (2013). 23986196 10.1152/ajpcell.00384.2012

[b47] Jung M., Lee J., Seo H. Y., Lim J. S. & Kim E. K. Cathepsin inhibition-induced lysosomal dysfunction enhances pancreatic beta-cell apoptosis in high glucose. PLoS One 10, e0116972 (2015). 25625842 10.1371/journal.pone.0116972 PMC4308077

