# Supplementary material for: Cathepsin S attenuates endosomal EGFR signalling: A mechanical rationale for the combination of cathepsin S and EGFR tyrosine kinase inhibitors
Source: Sci Rep. 2016 Jul 8;6:29256. doi: 10.1038/srep29256 (PMC4937378; doi:10.1038/srep29256)
Supplement: Supplementary Information [file srep29256-s1.pdf]

Supplementary Information

Cathepsin S attenuates endosomal EGFR signalling: A mechanical rationale for the combination of cathepsin S and EGFR tyrosine kinase inhibitors

Chien-Chang Huang, Cheng-Che Lee, Hsiao-Han Lin, and Jang-Yang Chang*

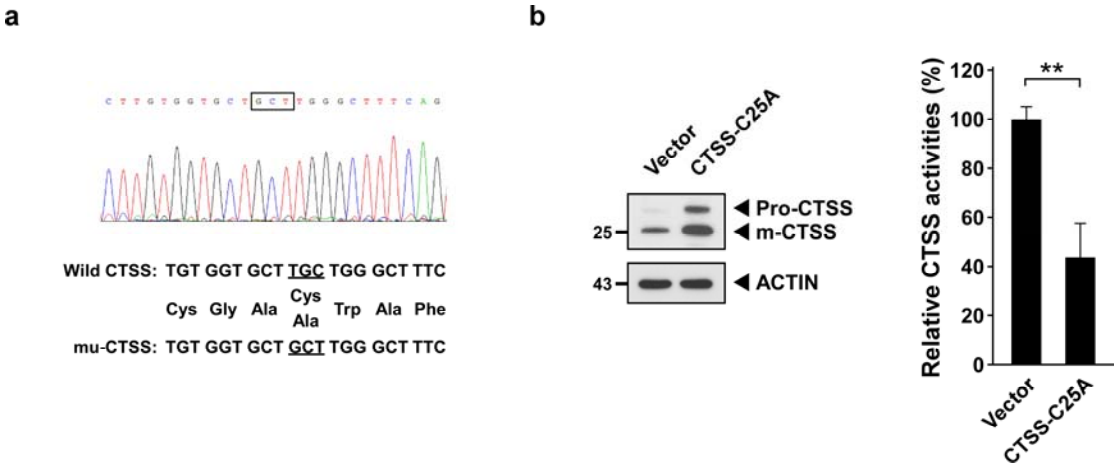

Figure S1. Total CTSS activities were reduced with CTSS-C25A mutant.

(a) The nucleotide sequences of CTSS inactive mutant C25A was validated and represented. (b) Left Panel: Overexpression of CTSS-C25A mutant was confirmed by Western Blotting analysis. ACTIN was used as the internal control for semiquantitative loading in each lane. Right Panel: Relative CTSS activities were examined with a fluorogenic cytochemical assay and quantified with a fluorescent microplate reader. The bars represent the relative CTSS activities of four independent wells ± SD. Differences were considered statistically significant at $^{**}P < 0.01$.

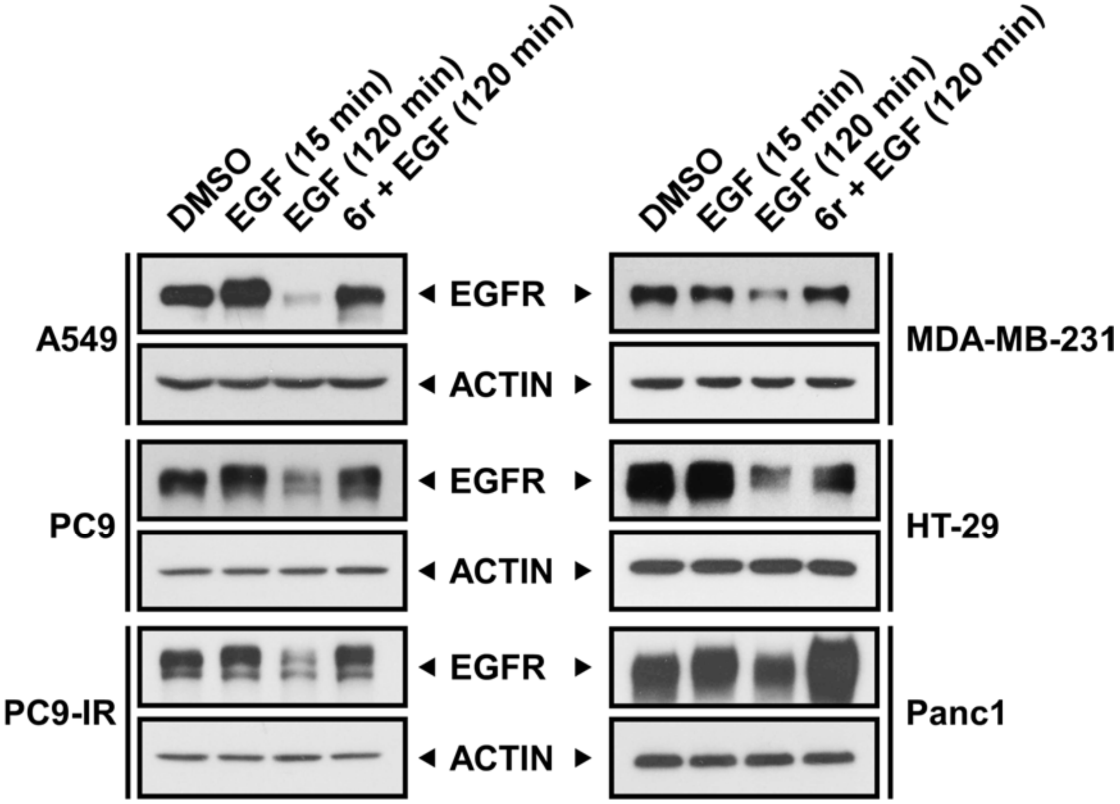

Figure S2. CTSS inhibition delayed EGF-mediated EGFR degradation process in various cell lines

Human non-small cell lung carcinoma A549, PC9, and PC9-IR, human breast cancer cell line MDA-MB-231, human pancreatic carcinoma Panc1, and human colon adenocarcinoma HT29 cell lines with and without the treatment of 20 μM 6r were stimulated with 100 ng/mL EGF for 2 h. Cell lysates were separated through SDS-PAGE and then probed with EGFR antibodies.
